# Use of Germline Genetic Variability for Prediction of Chemoresistance and Prognosis of Breast Cancer Patients

**DOI:** 10.3390/cancers10120511

**Published:** 2018-12-12

**Authors:** Viktor Hlavac, Maria Kovacova, Katerina Elsnerova, Veronika Brynychova, Renata Kozevnikovova, Karel Raus, Katerina Kopeckova, Sona Mestakova, David Vrana, Jiri Gatek, Pavel Ostasov, Radka Vaclavikova, Pavel Soucek

**Affiliations:** 1 Laboratory of Pharmacogenomics, Biomedical Center, Faculty of Medicine in Pilsen, Charles University, 323 00 Pilsen, Czech Republic; viktor.hlavac@lfp.cuni.cz (V.H.); katka.michalka@post.cz (K.E.); veronikabrynychova@seznam.cz (V.B.); rvaclavikova@seznam.cz (R.V.); 2 Third Faculty of Medicine, Charles University, 100 00 Prague, Czech Republic; maria.kovacova@lf3.cuni.cz; 3 Department of Oncosurgery, Medicon, 140 00 Prague, Czech Republic; renata.kozevnikovova@onko-centrum.cz; 4 Department of Breast Services, Institute for the Care for Mother and Child, 147 00 Prague, Czech Republic; karel.raus@quick.cz; 5 Department of Oncology, Second Faculty of Medicine, Charles University and Motol University Hospital, 150 06 Prague, Czech Republic; katerina.kopeckova@fnmotol.cz; 6 Department of Surgery, Second Faculty of Medicine, Charles University and Motol University Hospital, 150 06 Prague, Czech Republic; sonamuf@yahoo.com; 7 Department of Oncology, Palacky University Medical School and Teaching Hospital, 771 47 Olomouc, Czech Republic; davvrana@gmail.com; 8 Department of Surgery, EUC Hospital and University of Tomas Bata in Zlin, 760 01 Zlin, Czech Republic; gatekj@gmail.com; 9 Laboratory of Tumor Biology, Biomedical Center, Faculty of Medicine in Pilsen, Charles University, 323 00 Pilsen, Czech Republic; pavel.ostasov@lfp.cuni.cz

**Keywords:** breast cancer, chemoresistance, pharmacogenomics, next generation sequencing, in silico prediction

## Abstract

The aim of our study was to set up a panel for targeted sequencing of chemoresistance genes and the main transcription factors driving their expression and to evaluate their predictive and prognostic value in breast cancer patients. Coding and regulatory regions of 509 genes, selected from PharmGKB and Phenopedia, were sequenced using massive parallel sequencing in blood DNA from 105 breast cancer patients in the testing phase. In total, 18,245 variants were identified of which 2565 were novel variants (without rs number in dbSNP build 150) in the testing phase. Variants with major allele frequency over 0.05 were further prioritized for validation phase based on a newly developed decision tree. Using emerging in silico tools and pharmacogenomic databases for functional predictions and associations with response to cytotoxic therapy or disease-free survival of patients, 55 putative variants were identified and used for validation in 805 patients with clinical follow up using KASP^TM^ technology. In conclusion, associations of rs2227291, rs2293194, and rs4376673 (located in *ATP7A,* *KCNAB1,* and *DFFB* genes, respectively) with response to neoadjuvant cytotoxic therapy and rs1801160 in *DPYD* with disease-free survival of patients treated with cytotoxic drugs were validated and should be further functionally characterized.

## 1. Introduction

Breast cancer is the most frequent cancer in women worldwide [1]. The efficacy of breast cancer therapy is associated with a number of cellular processes that in some cases lead to tumor resistance. Among other factors, inactivation of anticancer drugs by biotransformation enzymes, decreased uptake and/or increased efflux of drugs, changes in cell-cycle checkpoints, increased DNA repair or reduced cell death, and cellular compartmentalization may contribute to the development of multidrug resistance [2].

The majority of currently used cytotoxic drugs are metabolized by biotransformation enzymes in liver and extrahepatic tissues. Biotransformation often leads to inactivation of drugs which become more polar to allow for body elimination. On the other hand, prodrugs are designed to be activated via biotransformation in the first place and then follow the same principles of metabolism as drugs. Consequently, germline genetic variability in biotransformation enzymes is considered as important factor determining individual patient sensitivity to an administered drug. These enzymes are in general divided into phase I (activation) and phase II (conjugation) enzymes [3]. Cytochromes P450 (CYP) constitute a major group of (in)activation enzymes in phase I whereas phase II enzymes are more heterogeneous. Numerous pharmacogenomic studies in breast cancer patients concentrated mostly on analysis of selected polymorphisms in single or several genes from all biotransformation phases (reviewed in [4]), but a comprehensive germline genetic variability screen of the majority of these enzymes in breast cancer patient cohorts is virtually missing.

Since drug efflux is mediated by membrane-bound ATP-binding cassette (ABC) transporters [5] and drug uptake is provided by solute carrier (SLC) transporters [6] it seems obvious that equilibrium of these exporters/importers is important for prediction of cancer drug resistance [7,8]. Indeed, comprehensive transcriptomic profiling studies demonstrated gene expression deregulations of a number of ABCs and SLCs between tumor and paired non-malignant tissues from patients with solid tumors, e.g., colorectal [9], breast [10], pancreatic [11,12], and ovarian [13] suggesting their potential role in cancer progression. Moreover, these studies revealed a number of associations between gene expression levels of transporters, therapy response and survival of the patients with implications for prognosis and individualized therapy.

Data from publicly available large-scale sequencing studies have shown that genetic alterations in drug targets, cell death and major cancer driving pathways, e.g., PI3K/AKT/MTOR or RAS/MAPK, and nuclear receptors can be found across all cancer types; however, at highly variable frequencies [14]. Very recently, highly frequent deleterious somatic mutations relevant for clinical management, including PIK3CA, RTK/RAS/MAPK and cell cycle pathway genes, were found in inflammatory breast cancer patients through next generation sequencing analysis [15].

Pharmacogenomics represents an important tool for personalized medicine. Two major types of studies may be found in the published literature dealing with the issue of genetic susceptibility and drug response in oncology. First, studies of germline genetic variation, mainly polymorphisms, in homogeneous groups of patients treated with defined drug regimens [16]. Second, in vitro screening of drug response in human cancer cell lines with well characterized somatic genetic profile also helped to elucidate the genetic background of chemoresistance [17,18]. However, recent data from analysis of sensitivity of 993 cell lines to 265 drugs show that germline genetic variability can be of the same importance as somatic one [19]. Thus, continuing with studies in patients is imperative for further understanding and subsequent translation of these aspects into clinical setting. There are several genome-wide association studies (GWAS) in the literature demonstrating the power of pharmacogenomics in breast cancer [20] and accelerated implementation of the next generation sequencing into clinical studies will undoubtedly bring further progress in this area. Very recently, analysis of available big data demonstrated that priority pharmacogenes for population-adjusted genetic profiling exist with highly variable distribution across populations [21], suggesting that use of sequencing-based approaches may enable “true personalized medicine”.

Here, we explored the genetic variability of a panel of 509 genes relevant for pharmacogenomics using targeted sequencing in a testing set of patients treated with neoadjuvant or adjuvant cytotoxic therapy. Genetic variants significantly associating with therapy outcome measured as clinical response in neoadjuvant setting or disease-free survival (DFS) of the patients were evaluated in a larger validation set of patients. To our knowledge, this is the first research study providing genetic data with association to drug chemoresistance evaluated as prognosis and therapy outcome of breast cancer patients with aid of in silico prediction in the Czech population to such an extent. The validated variants may further be used for functional studies and prospective follow up trials evaluating their prognostic and predictive utility in clinical setting.

## 2. Results

### 2.1. Testing Phase

The clinical characteristics of the patients in the testing set (*n* = 105) are shown in Table 1. Patients were treated with neoadjuvant cytotoxic therapy and/or with adjuvant therapy following surgical treatment. Cytotoxic therapy was based on monotherapy or combinations of anthracyclines, cyclophosphamide, 5-fluorouracil and taxanes (Table S1). The mean follows up of the patients was 70 ± 28 months. One patient was lost to follow up.

In these patients, a panel of 509 genes (Table S2) representing major drug metabolizing and transporting enzymes, nuclear receptors, cell death, chemotherapy target, and signaling pathway genes (Figure S1), selected using PharmGKB (www.pharmgkb.com) and Phenopedia (https://phgkb.cdc.gov) databases was assessed using targeted sequencing.

#### 2.1.1. Targeted Sequencing, Processing and Quality Control of Raw Data

**Figure 1:**
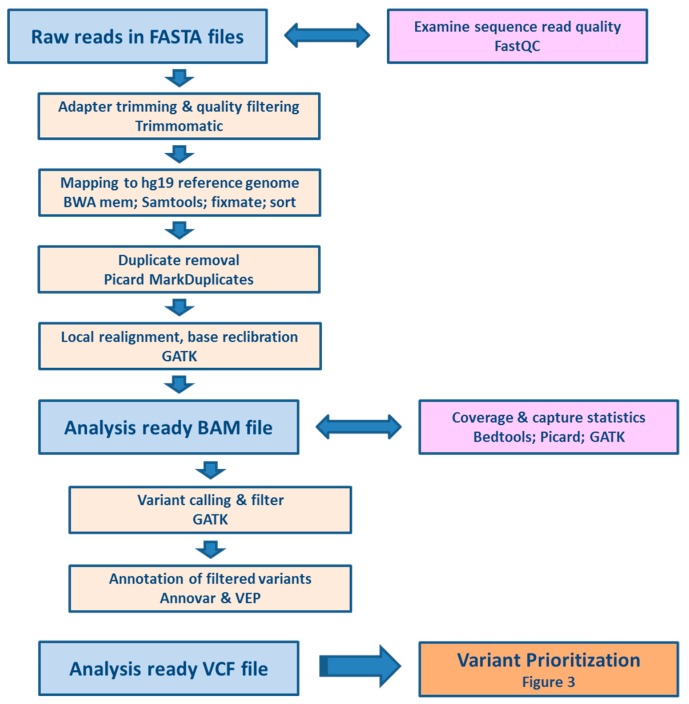
Pipeline for processing and quality control of raw sequencing data.

Processing of raw reads, quality control, filtering and annotation of the variants is depicted in Figure 1.

Quality control of the reads was performed in FastQC program and coverage was calculated after raw reads preprocessing (trimming and duplicate removal) by GATK 3.7. The average coverage was 76.9 ± 19.3 and 94% of the captured regions were covered at least 10 times. Altogether, we found 18,245 variants in exonic and adjacent intronic sequences.

Of the total number 509 genes, 503 genes (99%) contained at least one genetic alteration. No alterations were found in *ABCF1, HSPA1A, RXRB, TAP1 (ABCB2), TAP2 (ABCB3)* and *VDAC1P4* genes. On the other hand, the most polymorphic genes with over one hundred alterations were *NCOR2, ABCA13, RPTOR, ABCA4, CIT, BIRC6, ABCC1, ABCA1, RXRA, NCOR1, ABCA7, ABCC4* and *ABCB5*. Of the total number 18,245 variants, 3256 were in exons, 9458 intronic, and 3872 were in 3′UTR or 5′UTR regions according to NCBI Reference Sequence Database, RefSeq (RefSeq; https://www.ncbi.nlm.nih.gov/refseq/) in Annovar (Table 2).

7539 variants (41%) had minor allele frequency (MAF) > 0.05; the rest, 10,706 variants, had MAF 0.05 or below. On average, each patient showed 3792 ± 240 variants.

We found 88 loss of function truncating variants that were either affecting the stop codon (gain or loss) or frameshift insertions or deletions (indels). 1646 of the variants were non-synonymous single nucleotide variants (SNVs) and 1455 were synonymous SNVs (Table 3).

**Figure 2:**
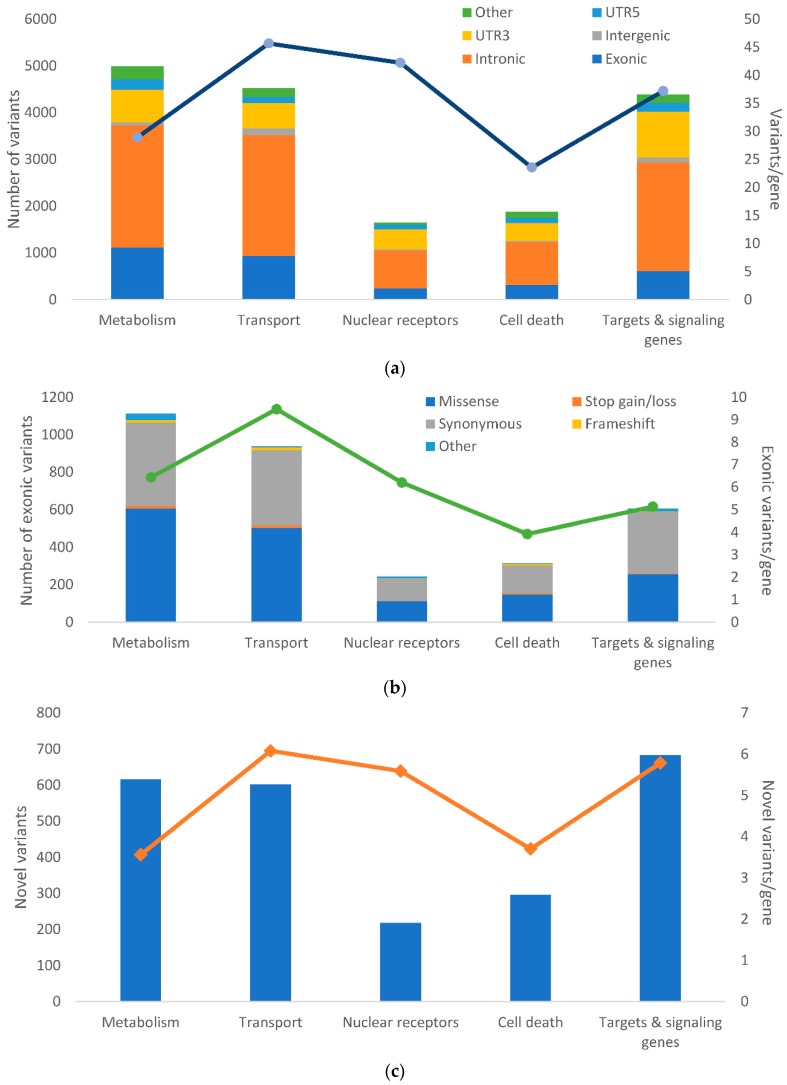
Distribution of alterations in the studied groups of genes. The picture shows: (**a**) the frequency of genetic alterations according to their functional classes; (**b**) The frequency of genetic alterations according to their exonic functional classes analyzed by RefSeq: NCBI Reference Sequence Database (https://www.ncbi.nlm.nih.gov/refseq/) shown according to the groups of studied genes; (**c**) The number of novel variants according to the groups of genes. The number of the variants normalized to the counts of genes per each group are shown for each plot on the right axis and depicted by the overlaid line. Column plots for all gene groups are shown in Figure S2.

Altogether, 2565 (14%) of the variants were novel (i.e., not found in dbSNP Build 150). The distribution of the variants and position in protein according to their functional classes and frequencies of novel variants in the groups of genes are shown in Figure 2.

#### 2.1.2. Prioritization of Variants for the Validation Phase

Variants with MAF > 0.05 were considered relevant to achieve adequate statistical power for variant interpretation. Variants that were not in Hardy-Weinberg equilibrium (*n* = 842) were excluded from analyses. In addition, variants with the missing data in more than 50% patients were excluded (*n* = 432). Further filtration parameters were applied (see Section 4 Materials and Methods) and these resulted in set of 5875 variants. In these variants, the associations with response of patients to neoadjuvant cytotoxic therapy and survival of patients were assessed in order to reveal genetic alterations with putative functional effect in vivo.

We found 327 variants (two novel) associated with the response to neoadjuvant cytotoxic therapy and 418 (three novel) variants associating with DFS (Table S3). Using Kaplan-Meier plots for variants associated with DFS, gene dosage relationship was evaluated. Those variants in which heterozygous genotype had the most pronounced effect (compared to both homozygotes) were excluded and a final set of statistically significant variants with clinical associations was built. The testing set was composed of both neoadjuvantly and adjuvantly treated patients. Therefore, we divided the testing set into two corresponding subsets and computed the DFS separately. The neoadjuvant subset (*n* = 68) comprised three molecular subtypes (luminal A, luminal B and triple negative; data were missing for nine patients) while the adjuvant subset (*n* = 37) comprised only patients with triple negative tumors. Due to this unevenness, we have also analyzed the associations of variants with molecular subtypes and displayed these for comparison in Table S3.

**Figure 3:**
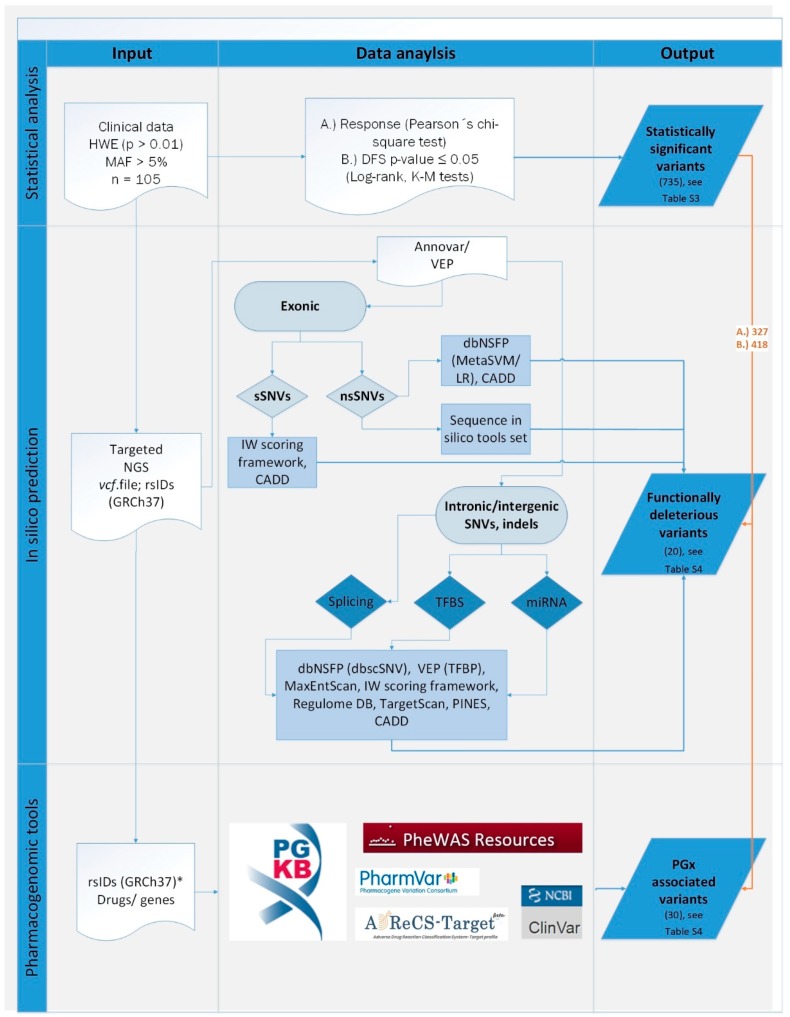
Variant prioritization scheme. Numbers of unique variants shared with statistically significant results are depicted in brackets. Statistical analysis using germline variants from targeted sequencing and clinical data of patients with Hardy-Weinberg equilibrium (HWE, *p* > 0.01) and Minor allele frequency (MAF > 0.05) was conducted to search for drug response (A) and (B) disease-free survival (DFS) associations. In silico prediction was applied on synonymous (sSNVs) and non-synonymous (nsSNVs) single nucleotide variants and indels from next generation sequencing (NGS) data in VCF format using several web-based and command-line software tools (see Section 4 Materials and Methods). In pharmacogenomic (PGx) databases with no batch or download option, only statistically significant variants were considered for manual curation (*). GRCh37 = Genome Reference Consortium Human Build 37 (hg19); TFBS = transcription factor binding site; TFBP = transcription factor binding profile.

In order to select the most relevant functional alterations from the statistically significant set of variants we down-sampled the results using information from in silico predictions and according to confirmed pharmacogenomic and clinical evidence. Annotations were conducted by Annovar and Variant Effect Predictor (VEP). Choice of in silico tools was based on the scope of the prediction for given software with the intention to ensure annotation for all types of coding and noncoding alterations (see Figure 3 and Section 4 Materials and Methods). Pharmacogenomic evidence was based mostly on PharmGKB database of published phenotypes (manual data curation is depicted in Figure S3). Variants with records in ClinVar indicating drug or any disease association were considered pathogenic. Additionally, in cases where information regarding drug response was available in ClinVar and/or dbSNP databases, a match with PharmGKB data provided an extent of evidence and level of priority. All variants significantly associated with response to neoadjuvant cytotoxic therapy of DFS were compared with records in these databases and variants were ordered by the level of priority (Table 4).

Following these priorities, 58 variants (56 SNVs and two indels; Table 5) were selected for validation in a larger cohort (*n* = 805) of breast cancer patients. The overall variant prioritization scheme is depicted in Figure 3.

### 2.2. Validation Phase

The clinical characteristics of the patients in the validation set (*n* = 805) are shown in Table 6.

Patients were treated with neoadjuvant cytotoxic therapy and/or with adjuvant therapy following surgical treatment. Cytotoxic therapy was based on the same drug combinations as in the testing set (Table S5). A small fraction of patients with localized disease and good prognosis was not treated with cytotoxic or hormonal therapy (*n* = 83). The mean follows up of the patients was 76 ± 30 months. Sixty patients were lost to follow up and could not be further evaluated in survival analyses.

All clinical characteristics were tested as modifiers of survival functions. High tumor grade (*p* = 0.008), advanced disease stage (*p* < 0.001), and the lack of expression of estrogen (*p* = 0.004) and progesterone (*p* = 0.013) receptors predicted worse DFS. Thus, these clinical factors were subsequently used for adjustment of multivariate survival analyses. Analogously, all clinical characteristics were tested on association with response. Only the advanced disease stage was a modifier of therapy response (*p* < 0.001) and was used for adjustment of multivariate analysis.

#### 2.2.1. Genotyping

Together, 58 variants were assessed by KASP^TM^ method in 805 DNA samples within the validation phase. Despite several attempts to optimize detection techniques, three variants (rs1065852, rs3815583 and rs3322) failed to perform consistently and could not be further evaluated for clinical associations. No variants significantly departed from Hardy-Weinberg equilibrium (*p* > 0.01). Out of theoretical 55 × 805 (44,275) data points, 257 (less than 1%) were missing due to uncertainty in genotype calling or absent signal. Highest missing data rate among individual variants was 30 (3.7%) and 800 samples had less than 10% of missing data. For 86 samples from the testing phase results of KASP^TM^ genotyping were available for validation of the sequencing results. Overall non-reference discordance rate across 55 × 86 (4730) data points was 2.3 with all but one mismatches in heterozygotes (*n* = 45). Also, the MAFs of variants in the validation set did not substantially differ from those observed in the testing set (Table 7).

#### 2.2.2. Clinical Associations

In order to validate clinical associations observed in the testing phase, variants were evaluated against response and survival of patients in the validation set. All homozygous genotypes observed in less than five patients were grouped with the corresponding heterozygous genotype for enhancing the statistical power of comparisons.

The variants that associated with response in both testing and validation phase are listed in Table 8 and thus these variants are considered validated variants with putative clinical importance.

**Figure 4:**
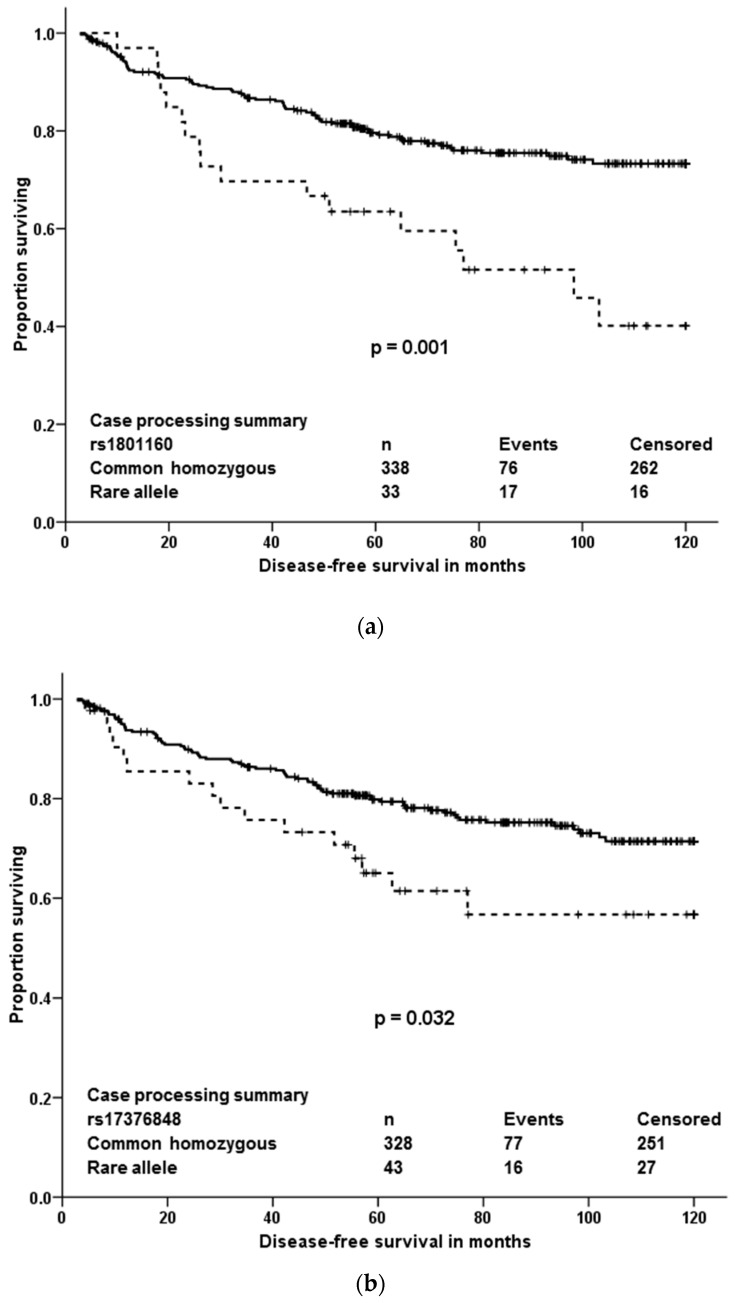

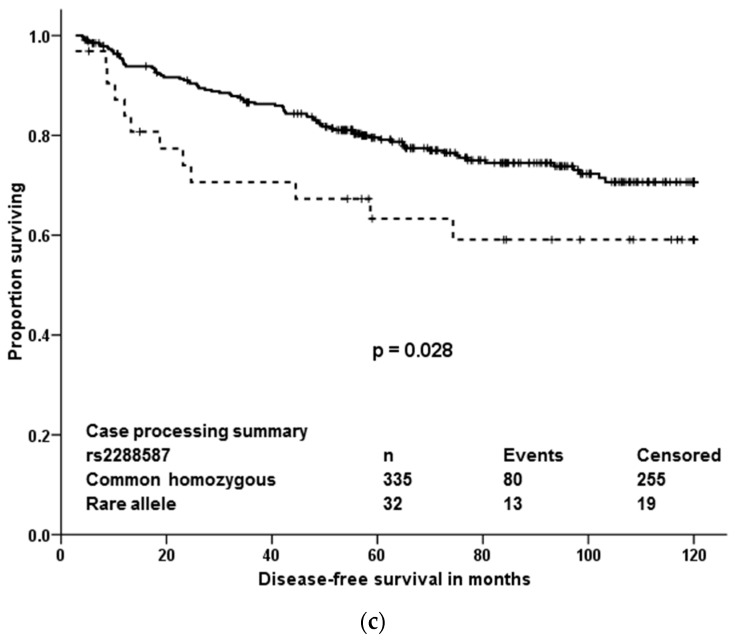
Kaplan-Meier plots with validated associations of variants with DFS of patients treated with cytotoxic therapy. Solid line represents the common homozygous genotype and dashed line the rare allele. Significance was evaluated by the log-rank test, *n* = number of individuals. In the absence of rare homozygotes in any of the compared groups, effect of rare allele was evaluated. (**a**): rs1801160; (**b**): rs17376848; (**c**): rs2288587.

Analogously to the testing phase, associations of variants with DFS of all patients or patients stratified according to therapy were evaluated. Two variants (rs12460651 and rs751141) significantly associated with DFS of all patients (*n* = 745; without 60 patients lost to follow up) and three other variants (rs17376848, rs1801160, and rs2288587) associated with DFS of patients treated with neoadjuvant and/or adjuvant cytotoxic therapy (*n* = 371; without six patients lost to follow up). These patients comprised molecular subtypes: luminal A (*n* = 101), luminal B (*n* = 166), HER2 (*n* = 36) and triple negative (*n* = 66); data were missing for two patients. Variant rs2075061 associated with DFS only in patients treated with hormonal therapy without cytotoxic drugs (*n* = 312. Luminal A, *n* = 187; luminal B, *n* = 118 and seven missing). Of these variants, rs12460651, rs2075061, and rs751141 did not pass the gene dosage condition (Figure S4) and thus these variants could not be further considered validated. Validated variants associating with DFS in the cytotoxic therapy treated patients are depicted in Figure 4. In order to clarify the effect of molecular subtype on prognosis of the patients treated with neoadjuvant and/or adjuvant cytotoxic therapy, these patients were further stratified according to their molecular subtype. Associations with DFS were then calculated separately for each subtype (Table 9 and Figure S5). If single *p*-value of stratified patients was delivered (pooled log-rank test), variants rs1801160 and rs2288587 remained significantly associated with DFS (*p* < 0.001 and *p* = 0.030, respectively), but rs17376848 was non-significant (*p* = 0.083).

In multivariate analyses, using Cox regression adjusted to tumor grade, disease stage and expression of hormonal receptors, the rare allele in rs1801160 in *DPYD* was associated with a significant hazard ratio (HR = 2.58, 95% CI = 1.48–4.50, *p* = 0.001), but the other two variants (rs17376848 also in *DPYD* and rs2288587 in *IRS1*) were non-significant (*p* = 0.071 and *p* = 0.115, respectively).

## 3. Discussion

There is no doubt that drug therapy tailored to individual genetic predisposition could bring substantial cost-benefit effects in terms of both enhanced drug efficacy and decreased risk of adverse drug reactions. Pharmacogenomics seem so far instrumentally more accessible than routine pharmacokinetics or pharmacodynamics in clinical day use. However, current approaches, including the state-of-the-art technological platforms such as the next generation sequencing, are still in the early evolutionary phase. Except the considerable decrease of cost per genotype in the last few years, the complexity of data management and the need for robust evaluation of results to make them clinically meaningful still hinder broader usage, especially in the pharmaceutical area. Thus, studies addressing these aspects are urgently needed.

The present paper shows that out of quite large number of germline variants (18,245) detected among 509 pharmacogenes and other drug-related genes in breast cancer patients, only a few may be important from the view of individualized therapy after robust validation. Four variants associated with response of patients to neoadjuvant cytotoxic therapy and three, out of which just one was significant in multivariate analyses, were prognostic in terms of DFS after cytotoxic therapy. Responders to the neoadjuvant cytotoxic therapy carried, significantly more frequently than non-responders, the rare allele in rs10868138 of *SLC28A3*, the common homozygous genotype in rs2227291 (*ATP7A*), or rs4376673 (*DFFB*) or the common allele in rs2293194 (*KCNAB1*). However, the association of rs10868138 with response should be treated with caution while this association was non-significant after adjustment for disease stage in multivariate analysis. Rs2293194 was significantly associated with response only in patients with early stage disease 0 or I (*p* < 0.001). Patients with the common homozygous genotype in rs1801160 of *DPYD* survived longer without relapse after cytotoxic therapy than carriers of the rare allele. This effect was particularly pronounced in patients with luminal B and triple negative molecular subtypes.

Protein coding gene *SLC28A3* (Solute carrier family 28 member 3) is a sodium-dependent nucleoside transporter involved in the homeostasis of endogenous nucleosides and regulating multiple cellular processes, e.g., neurotransmission and metabolism and transport of nucleoside drugs [22] (https://www.genecards.org/cgi-bin/carddisp.pl?gene=SLC28A3&keywords=A-3). Genetic variability in *SLC28A3* was previously connected with pharmacokinetics of nucleoside analogs [23] and cardiotoxicity of anthracyclines [24] although a more recent study did not confirm the latter observation [25]. Variation rs7867504 in *SLC28A3* was shown to be involved in gemcitabine pharmacobiology and toxicity in metastatic breast cancer patients receiving maintenance therapy [26] or in patients with pancreatic carcinoma [27]. The rs10868138 in *SLC28A3* is a less studied variant; however, it was recently connected with higher concentration of azathioprine in erythrocytes of patients with neuromyelitis optica [28], suggesting that it may be functional in vivo. The other relevant gene to pharmacogenomics of nucleoside analogs is dihydropyrimidine dehydrogenase (*DPYD*). The protein encoded by this gene (DPD) is a pyrimidine catabolic enzyme and the initial and rate-limiting factor in the pathway of uracil and thymidine catabolism (https://www.genecards.org/cgi-bin/carddisp.pl?gene=DPYD). DPD is active in the catabolic pathway of 5-fluorouracil and mutations in its gene result in an increased risk of toxicity in cancer patients receiving 5-fluorouracil chemotherapy [29]. *DPYD* polymorphism rs1801160, associated with survival of breast cancer patients in our study, is very frequently studied. Carriage of rs1801160 in *DPYD* associated with grade 3 or 4 5-fluoropyrimidine associated adverse risk effects, e.g., neutropenia, in a recent study of colon cancer patients treated with regimens consisting of 5-fluoro-uracil or capecitabine combined with oxaliplatin [30]. Although *DPYD* genotype-guided individualized dosing for better safety of fluoropyrimidine treatment was recently suggested as a new standard of care [31], the present study was not designed to address adverse effects and the validated association of *DPYD* variant with DFS adds a new observation to the knowledge base.

Of the other validated variants associated with response to the therapy, the rs2227291 in *ATP7A* raises particular attention since it is non-synonymous (V767L) and thus probably more directly functional. Notably, rs2227291 is the only association with response in the validation set that passes the false discovery rate (*p* = 0.011). Copper transporter ATP7A encodes a transmembrane protein that functions in copper transport across membranes and it is frequently studied in connection with sensitivity to platinum drugs, e.g., cisplatin. Very recently, the rs2227291 was associated with cisplatin resistance in patients with epithelial ovarian cancer treated with combination of platinum and taxane [32]. However, though the authors state that carriers of the minor allele are more sensitive to cisplatin, the functional link is missing and must be obtained using further study. GWAS studies show that regulatory non-coding variants may play a role in multiple distinct diseases such as cancer [33] and thus the other two intronic variants associated with response (rs2293194 in *KCNAB1* and rs4376673 in *DFFB*) also represent a potential target for further studies. *KCNAB1* (Potassium Voltage-Gated Channel, Shaker-Related Subfamily, Beta Member 1) gene encodes a potassium channel involved in an important dopamine pathway, chemical transmission of signal across synapses and various CYP450 pathways (https://www.genecards.org/cgi-bin/carddisp.pl?gene=KCNAB1). In cancer genetics, *KCNAB1* variation may play a role in breast cancer pathogenesis because its overexpression was found in breast tumors in comparison to non-tumor tissues [34]. Finally, *DFFB* is a subunit Beta and active component of DNA Fragmentation Factor protein (DFF). *DFFB* has been found to trigger both DNA fragmentation and chromatin condensation during the apoptosis (https://www.genecards.org/cgi-bin/carddisp.pl?gene=DFFB). For example, enhanced expression of DFFB with doxorubicin or in combination with sulfonamides enhanced the killing of T47-D breast cancer tumor cells via apoptosis [35,36]. Thus, variation and deregulation of the DFFB gene in the presence of apoptosis-inducing drugs might have an impact on their efficiency in tumor cells.

Of the 88 loss of function variants identified in our study, only seven frameshift variants had MAF above 5% ensuring the necessary statistical power to estimate the associations with DFS or response. None of the associations of frameshift indels with outcome was statistically significant. Of the genes harboring these variants, only *RRM2B*, was in the first quartile of the most intolerant genes to functional variation, according to LoFtool gene score [37]. The rest of the genes in the first quartile were *ABCA5/A6/A7/A10/A13*, *ABCB4/B10*, *ABCC2/C3/C5/C10/C11/C12*, *DHCR7*, *NR1I3*, *SLC35C2* and *SLCO3A1*. Of their corresponding proteins, mainly the multidrug resistance protein (MRP)2, MRP3, MRP5, and MRPs 7–9 coded by membrane transporters *ACBC2*, *ABCC3*, *ABCC5*, *ABCC10*, *ABCC11*, *ABCC12* and the organic anion transporter polypeptide-related protein (OATP)3A1 coded by *SLCO3A1* are of the highest importance because of the relation of MRPs and OATPs in the chemotherapy resistance or sensitivity [5,6]. However, associations with response or DFS in these genes could not be assessed due to the modest size of our cohort and the low frequency of these variants in population.

Population context is currently broadly discussed, for example considerable gene-dependent variability between African and European Americans has recently been demonstrated [21]. The present study was performed on homogeneous population of Slavic Europeans. As such, adds unique information to the existing clinically associated datasets. The only publicly available data in the Czech population on the germline whole exome level are in the National Center for Medical Genomics (NCMG) set of healthy Czech population (*n* = 309 at time of writing). Of the total number of 509 genes, 503 genes (99%) contained at least one genetic alteration. No alterations were found in *ABCF1, HSPA1A, RXRB, TAP1 (ABCB2), TAP2 (ABCB3)* and *VDAC1P4* genes in the present study. However, in comparison to the data in the NCMG set of healthy Czech population, these genes, except *VDAC1P4,* were polymorphic. In total, 54 variants were found in *ABCF1*, 13 in *HSPA1A*, 16 in *RXRB*, 78 in *TAP1*, and 88 in *TAP2*. Whether these differences are due to the different composition of both sets in terms of individual characteristics of participants (the present set contained only females while the NCMG set is composed of both genders) or due to the disease etiology (breast cancer patients *versus* healthy population) remains to be elucidated. Differences between sequencing platforms, raw data management and annotation cannot be excluded either. On the other hand, the most polymorphic genes with over one hundred alterations were *NCOR2, ABCA13, RPTOR, ABCA4, CIT, BIRC6, ABCC1, ABCA1, RXRA, NCOR1, ABCA7, ABCC4* and *ABCB5* in the present study and except for *RXRA,* all these genes showed more than 100 alterations in the NCMG set as well. *ABCA13* is overall the 80th most polymorphic gene in NCMG data coming from the whole exome sequencing, while the rest of the top 80 variable genes in NCMG data were not analyzed in this study. Thus, although there are some similarities in these sets, the direct comparison of data from two sets within the same population points to some differences, mainly in the low MAF variants, and thus, multiethnic cohorts must be very carefully evaluated in this regard. The recent study by Kozyra et al. [21] reported *ABCA4, ABCA1* and *ABCC1* among genes with highest counts of variations suggesting that the most variable genes may be conserved across diverse populations.

We aimed not only to contribute to the search for predictive genetic biomarkers for oncology, but also to set up a pipeline for processing of raw data generated by massively parallel gene panel sequencing, including quality controls. Last but not least, complex variant prioritization scheme including both evaluation of variants by associating them with individual patient data relevant to their pharmacological response and further filtration using in silico predictive tools and pharmacological databases is provided. Moreover, robust validation by means of comparison of results obtained by two technological platforms and two stage study evaluation using testing and validation clinical cohorts was accomplished.

Public databases such as PharmGKB are wealthy sources of germline variants which evolved from laborious curation of published studies and a strong need for systematic use of perished knowledge in personalized medicine [38,39]. At the time of writing of this article, 21,115 annotations in 647 drugs associated with drug response at pharmacodynamic and/or pharmacokinetic level were in PharmGKB (https://www.pharmgkb.org/, accessed 4 November 2018). Despite the significant number of annotations available, automated prediction for drug response of sequenced variants is not available. Many in silico tools have been developed to aid with the prediction mostly for coding variants. Evolutionary characteristics of variants in pharmacogenes involved in biotransformation and transport of drugs are, however, different. This complicates accurate estimates provided by methods mostly built on Mendelian disease principles [40]. Consequently, genomic evolutionary rate profiling or evolutionary constraint algorithms, as well as tools trained on disease pathogenic/neutral variants were not included in our in silico sequence tools set. Several attempts have been made to generate specialized tools scaled for pharmacogenes or to optimize current models for pharmacogenetic assessments [40,41]. Nonetheless, “gold standard” methods are still lacking in the public domain. Furthermore, even no standard recommendations on the number or types of in silico tools to be considered in analyses which may have significant impact on results are available [42]. While this prevents to a certain extent potentially incorrect use and interpretation in clinical practice, academic research is also hindered. In our research we attempted to combine different approaches to acquire complex information for given variants. Prediction or knowledge acquired for prioritized variants was not further manually curated. The reason was to verify the ability of automated prioritization and to estimate the added value of in silico tools for our further studies.

The modest size of the testing set may be seen as a limitation of the study. Due to this fact, the importance of very rare (MAF < 0.001) and rare (<0.01) variants could not be assessed. Thus, we prioritized variants with MAF > 0.05. In the light of the recently acknowledged contribution of rare variants to inter-individual variability in drug response [40] this limitation needs to be considered in future pharmacogenomic studies in oncology. On the other hand, ethnical homogeneity and completeness of clinical follow up is considered beneficiary. Moreover, the study may be extended by addition of more patients or compiling with similarly designed set of patients with whole exome or genome data. Another limitation of this study is the nonhomogeneity of the patient sets. The advanced disease stage and the molecular subtype can be seen as the strongest modifiers of patient prognosis. We have employed the multivariate analyses adjusted to disease stage and we have analyzed the associations of variants with molecular subtype in the testing set to circumvent these issues. We also analyzed associations with survival separately in neoadjuvantly treated patients (with predominant luminal subtype) and adjuvantly treated patients (triple negative tumors only) in the testing set and ran the full prioritization pipeline again. Despite some slight discrepancies which might be caused by chance due to small sizes of compared groups, all the major conclusions of this study remained unchanged.

Functional studies of the identified variants and genes will be the next step. Functionality of a variant may be studied using CRISPR-Cas9 gene editing in a suitable tumor cell model *in vitro.* Subsequently gene function, including response of the model cell line to clinically relevant drugs, e.g., taxanes, may be followed.

## 4. Materials and Methods

### 4.1. Patients

The testing study included a total of 105 breast cancer patients of Caucasian origin diagnosed in the Institute for the Care for Mother and Child and Medicon in Prague and Hospital Atlas in Zlin (all in the Czech Republic) during 2006–2013. Patients underwent neoadjuvant cytotoxic therapy with regimens based on 5-fluorouracil/anthracyclines/cyclophosphamide (FAC or FEC) and/or taxanes (*n* = 68) or postoperative adjuvant therapy using the same cytotoxic drugs (*n* = 37). The validation set was composed of 805 breast cancer patients recruited in Motol University Hospital, Institute for the Care for Mother and Child, and Medicon in Prague and Hospital Atlas in Zlin during 2001–2013.

Collection of blood samples and retrieval of clinical data was performed as described previously [43]. The following data on patients were retrieved from medical records: age at diagnosis, menopausal status, personal medical history, family history (number of relatives affected by breast/ovarian carcinoma or other malignant diseases), stage, tumor size, presence of lymph node metastasis, histological type and grade of the tumor, expression of estrogen, progesterone, and ERBB2 (v-erb-b2 avian erythroblastic leukemia viral oncogene homolog 2, OMIM:164870) receptors, expression of Ki67 (proliferation-related Ki-67 antigen, OMIM:176741), response to the therapy according to RECIST criteria [44], and DFS. DFS was defined as the time elapsed between surgery and disease recurrence. Response to the neoadjuvant therapy was evaluated based on ultrasonography performed before and after the cytotoxic therapy.

All patients were informed about the study and those who agreed and signed an informed consent participated in the study. The study was approved by the Ethical Commission of the National Institute of Public Health in Prague (ethic code: Č.15-25618A, 6 August 2014). The methods were carried out in accordance with guidelines approved by the Ethical Commission.

### 4.2. DNA Extraction

Blood samples were collected during the diagnostic procedures using tubes with K3EDTA anticoagulant. Genomic DNA was isolated from human peripheral blood lymphocytes by the standard phenol/chloroform extraction and ethanol precipitation method [45]. DNA was quantified by Quant-iT PicoGreen DNA Assay Kit (Invitrogen, Carlsbad, CA, USA). DNA samples were stored in aliquots at −20 °C prior to analysis.

### 4.3. Gene Panel Selection

The genes were selected according to the following criteria: (i)gene in PharmGKB with published association to anthracyclines, doxorubicin, daunorubicin, 5-fluorouracil, cyclophosphamide, paclitaxel or docetaxel; pharmacokinetics pathway of doxorubicin, 5-fluorouracil, cyclophosphamide, docetaxel or paclitaxel; (ii)association with metabolism of xenobiotics, drug metabolism, sulfur metabolism and transport in Phenopedia. The selected genes were divided into groups according to major function, i.e., transport (ABCs, SLCs), metabolism (CYPs, UGTs, etc.), cell death (CASPs, BCLs, etc.), nuclear receptors, targets and signaling genes (Figure S1). All selected genes were validated using NimbleDesign web tool (Nimblegen, Roche, Prague, Czech Republic) (list of genes in (Table S2).

### 4.4. Targeted Sequencing

Libraries encompassing all exons of selected genes were prepared following the previously published design [46]. Based on the character of probe design, i.e., tiling; the exons were surrounded by approximately 30 bp regions of intronic sequences which were also sequenced in both directions. Target enrichment was performed by the Nimblegen’s SeqCap EZ Choice (Roche, Prague, Czech Republic) using a standard SeqCap protocol [47]. Samples were sequenced on an Illumina MiSeq platform (Illumina Inc., San Diego, CA, USA). Twelve samples were sequenced in each run with the planned minimal coverage 60–100. Data were aligned to the hg19 reference genome with the Burrows-Wheeler Aligner (BWA, Cambridge, UK) 0.7.12 [48], SAM to BAM conversion was done by Samtools 1.4.1 (Wellcome Trust Sanger Institute, Hinxton, UK), duplicate removal by Picard 2.17.10 and base recalibration, local realignment and detection of single nucleotide polymorphisms (SNP) and small indels were done by the Genome Analysis Toolkit (GATK, Broad Institute, Cambridge, UK) 3.7 Haplotype Caller according to GATK Best Practices [49]. The variants were annotated using Annovar (version 2018 Apr 16, Pennsylvania, PA, USA) [50] and VEP 94 (Wellcome Trust Sanger Institute, Hinxton, UK) [51] (Figure 1).

### 4.5. Genotyping

In the validation phase, 58 genetic variants with clinical associations were analyzed in DNA from 805 breast cancer patients using KASP^TM^ technology (LGC Genomics, Hoddesdon, UK). Quality control was performed by determination of duplicate samples for approximately 10% of the samples in both phases. The genotyping concordance between duplicate samples exceeded 99%.

### 4.6. Data Analysis

The raw variants from targeted sequencing were recalibrated using GATK 3.7. Hardy-Weinberg test was computed using Bcftools 1.5 (Cambridge, UK). Only variants in Hardy-Weinberg equilibrium (*p* > 0.01), with MAF > 0.05 and with less than 50% of missing data were considered for statistical and functional evaluations. Comparison of response to the therapy with respect to groups of patients (common homozygous, heterozygous and rare homozygous) was based on the Pearson chi-square test for each variant. Adjusted *p*-value was calculated for each variant and each of these tests. Computation of adjusted *p*-value was as follows: (1) *p*-value based on original data was calculated; (2) 1000 permutations of original data were generated; (3) value of test statistic was calculated for each permutation; (4) proportion of *p*-values based on permuted data (1000 *p*-values for each test) which were higher or equal than *p*-value based on original data was calculated; and (5) adjusted *p*-value was obtained for given variant. For multivariate analyses, binary logistic regression was used.

Comparison of DFS with respect to groups of patients (common homozygous, heterozygous and rare homozygous) was performed by the log-rank test for each variant separately. Kaplan-Meier plot for each variant separately was generated as well for visual comparison. As study follow-up was set to 120 months (10 years) then if the value of DFS for some subject was higher than 120 months, value for this subject was set to 120 and censored. Adjusted *p*-value for log-rank test was based on 100 permutations of original data. Methodology of computation for adjusted *p*-value for each variant was performed in a similar way as it was mentioned previously. A *p*-value of less than 0.05 after adjustment for multiple testing by using 100 permutations for survival and 1000 permutations for response was considered statistically significant. Analyses were conducted using the statistical program SPSS v16.0 (SPSS, Chicago, IL, USA) and using the R script. Cox regression was also performed in analysis of DFS separately for variants which were statistically significant based on adjusted *p*-values. Response variable was DFS and predictor variable was variant. Based on likelihood ratio test for testing statistical significance of variant, *p*-value was recorded. After that, adjustment on multiplicity only for these *p*-values was performed. Considered adjustments were Bonferroni method, Hochberg method, Hommel method, Holm method and false discovery rate method.

Analysis for comparison of DFS with respect to groups of patients was also performed for subgroups of adjuvantly treated patients and neoadjuvantly treated patients. Methodology for adjusted *p*-values derivation, Cox regression analysis and adjustment methods on multiplicity are the same as previously. Permutations of original data (100 permutations) were generated separately for adjuvant patients and for neoadjuvant patients.

For functional prediction germline variants were annotated by Annovar and VEP. Choice of in silico tools was based on scope of the prediction for given software with the intention of ensuring prediction for all types of consequences in our set. For non-synonymous/missense and splice site variants, dbNSFP 3.5a provided binary predictions by ensemble scores metaSVM, metaLR and dbscSNV scores, respectively [52]. In addition, a strict consensus in prediction results while excluding all variants for any of the missing prediction was applied to missense variants in “sequence in silico tools set”. The set encompass Mutation Assessor (H/M = functional), SIFT (D: Deleterious; ≤0.05), LRT (D: Deleterious) and Provean (D: Damaging) software tools. This approach is not based on machine learning methods and thus overcomes the concern of type 2 circularity due to insufficient discrimination of deleterious variants from neutral ones within given protein in training dataset [53]. CADD v1.3 (cut-off value ≥ 19, VEP) for all types of variants (i.e., coding, non-coding SNVs and short insertion/deletions) provided supplementary ensemble score. Further, splicing defect prediction was also annotated by VEP, flagging variants in a high information position of a transcription factor binding profile (TFBP). Splice donor/acceptor variants were annotated by MaxEntScan (based on the Maximum Entropy principle and neural networks) with score for reference and alternative variants. The higher score in MaxEntScan implied for a higher probability of the sequence being a true splice site. Known regulatory elements in the intergenic regions (e.g., DNAase hypersensitivity, binding sites of transcription factors, and promoter regions that have been biochemically characterized to regulation transcription) were predicted for deleteriousness by Regulome DB score [54] following classification category 1. These variants were considered likely affecting binding to transcription factors and expression of a gene target. A very recently developed web-based IW-Scoring framework (http://www.snp-nexus.org/IW-Scoring/) and PINES (Phenotype-Informed Noncoding Element Scoring) [55] were additionally used. IW score was provided for known (IW score K11) and novel (IW score N8) non-coding and coding synonymous variants. PINES (*p*-value ≤ 0.05) provided a ranked list of non-coding variants with functional characterization for liver tissue as a major site of drug biotransformation. Biological targets of miRNAs and conserved sites of given variant (UTR3) were matched by TargetScan (release 7.2, Cambridge, MA, USA).

Complementary to in silico predictions, evidence from pharmacogenomic and clinical databases was provided. Queried databases included, e.g., PharmGKB [38], PharmVar (available only for CYP2C9, 2C19, 2D6 genes), ADReCS-Target an Adverse Drug Reaction Classification System-Target Profile (http://bioinf.xmu.edu.cn/ADReCS-Target) [56], which provides comprehensive information about ADRs caused by drug interaction with protein, gene and genetic variation, PheWas Resources (phenome-wide association studies with antineoplastic drugs), Clinvar and dbSNP.

## 5. Conclusions

Through massive parallel sequencing, germline variability within a panel consisting of genes with relationships to drug metabolism and disposition, cell death and major oncogenic pathways was assessed in Czech breast cancer patients for the first time. Technically and clinically validated associations of rs2227291 in *ATP7A*, rs2293194 in *KCNAB1* (in early stage patients), and rs4376673 in *DFFB* with response to neoadjuvant cytotoxic therapy provide new putative loci for subsequent functional studies. The frequently studied rs1801160 in *DPYD* significantly associated with disease-free survival of patients treated with cytotoxic drugs and represents additional provocative target with prognostic potential namely in patients with luminal B or triple negative tumors.

Additionally, the present study brings complex insights into the prioritization of variants using individual clinical data, emerging in silico tools and established pharmacogenomic databases.

## Figures and Tables

**Table 1:** Clinical data of patient in the testing set.

Characteristics	Patients, *n* (%) ^1^
Age at diagnosis, mean ± S.D. ^2^ (years)	51.7 ± 9.4
Menopausal status	
Premenopausal	46 (46)
Postmenopausal	55 (55)
Missing data	4
Tumor size (pT)	
pTis	8 (8)
pT1	50 (48)
pT2	40 (39)
pT3	5 (5)
pTX	2
Lymph node metastasis (pN)	
Absent (pN0)	68 (65)
Present (pN1–3)	37 (35)
Pathological stage	
SI	46 (44)
SII	47 (45)
SIII	12 (11)
Histological type	
Invasive ductal carcinoma	88 (84)
Other type	17 (16) ^4^
Pathological grade (G)	
G1	11 (11)
G2	35 (35)
G3	54 (54)
GX	5
Estrogen receptor status	
Positive	38 (38)
Negative	61 (62)
Missing data	6
Progesterone receptor status	
Positive	39 (39)
Negative	60 (61)
Missing data	6
Expression of HER2	
Positive	2 (2)
Negative	97 (97)
Missing data	6
Expression of Ki-67, mean ± S.D. ^2^ (%)	32.9 ± 20.3
Molecular subtype	
Luminal A	15 (16)
Luminal B	23 (24)
Triple negative	58 (60)
Missing data	9
Response to neoadjuvant cytotoxic therapy	
Complete or partial response	47 (69)
Stable disease or progression	21 (31)
Not applicable ^3^	37

Footnotes: ^1^ Number of patients with % in parentheses; ^2^ S.D. = standard deviation; ^3^ patients treated with adjuvant therapy without neoadjuvant cytotoxic therapy; ^4^ six lobular, six medullary, two metaplastic, one mucinous, one papillary, and one neuroendocrine invasive carcinomas.

**Table 2:** Overview of the observed alterations in breast cancer patients by function according to Annovar.

Type	Total	Percentage
Downstream ^1^	353	1.9
Exonic (coding)	3256	17.8
Intergenic	372	2.0
Intronic	9458	51.9
Splicing ^2^	40	0.2
Upstream ^1^	414	2.3
UTR3	3106	17.0
UTR5	766	4.2
Other	480	2.7

Footnotes: ^1^ Variant is within 1 kb region downstream/upstream of transcription end site; ^2^ Variant is within 2 bp of a splicing junction.

**Table 3:** Overview of the observed exonic alterations in breast cancer patients by coding consequence.

Classification	Count	Percentage
Frameshift deletion	24	0.7
Frameshift insertion	22	0.7
Non-frameshift deletion	17	0.5
Non-frameshift insertion	8	0.2
Non-synonymous SNV	1646	50.6
Stopgain	40	1.2
Stoploss	2	0.1
Synonymous SNV	1455	44.7
Unknown	42	1.3

**Table 4:** Priorities and data input used for prioritization of variants for the validation phase.

Data Input	Priority			
Variant functionality	Highest	High	Medium	Low
Response or disease-free survival	significant	significant	significant	significant
PharmGKB	associated	associated	no data	no data
ClinVar	drug response or cancer/neoplasm	no data	drug response or cancer/neoplasm	no data
In silico prediction call	deleterious	deleterious/neutral	deleterious	neutral
Cancer related functionality	no	no	no	yes

**Table 5:** Prioritized variants for the validation phase.

Gene	HGVS Coding	HGVS Protein	Classification in Annovar ^1^	Rs ID	ClinVar	DFS ^2^	Response ^2^	Function ^3^	AF ^4^	ExAC ^5^	NCMG ^6^
*ABCA4*	c.5603A>T	p.N1868I	NS	rs1801466	likely benign	0.03		D; CADD	0.105	0.066	0.065
*ABCB1*			intronic	rs2032583		0.03		PA166157317	0.106	0.123	0.093
*ABCB5*			intronic	rs11983326		0.04	0.038	DFS & response	0.279	0.297	0.246
*ABCB8*			intronic	rs3214587		0.01		miR-670-3p	0.115		
*ABCC1*	c.*1512T>C		UTR3	rs212091		0.05		PA166154987	0.180		0.114
*ABCC1*	c.*543C>T		UTR3	rs3743527		0.05		PA166155049	0.205		
*ABCC3*			intronic	rs4148413			0.002	1f, PINES	0.168		
*ABCC6*	c.2835C>T	p.P945P	synonymous	rs2856585	pathogenic	0.03		ClinVar	0.064	0.099	0.044
*AHRR*			intronic	rs2013782		0.02		1f	0.587	0.623	0.597
*AKR7A2*	c.424G>A	p.A142T	NS	rs1043657		0.01		PA166161794; CADD	0.095	0.093	0.081
*AKT1*			intronic	rs3803304		0.05		PA166154802; 1f	0.292		0.289
***ATP7A***	**c.2299G>C**	**p.V767L**	**NS**	**rs2227291**	**benign**		**0.003**	**PA166157866**	**0.260**	**0.217**	**0.225**
*BAK1*	dist = 114		downstream	rs210134			0.033	1f, PINES	0.750		
*BIRC7*	c.528C>T	p.S176S	synonymous	rs2273487		<0.001		1b	0.486	0.467	0.464
*BLK*	c.-53667C>T		UTR5	rs922483	benign		0.023	1f	0.229		
*CDA*	c.79A>C	p.K27Q	NS	rs2072671		0.01		PA166153667	0.362	0.343	0.296
*CES1*	c.-75T>G		UTR5	rs3815583			0.038	PA166155058	0.202	.	0.162
*CES1*	c.224G>A	p.S75N	NS	rs2307240		0.01		PA166155039	0.067	0.054	0.063
*CES1*			intronic	rs76336259		0.001	0.046	DFS & response	0.063		0.060
*CMPK1*	c.22G>C	p.G8R	NS	rs7543016		0.05		PA166153793	0.451	0.538	0.320
*CYP2C9*			intronic	rs1934969		0.03		PA166153986	0.613		0.658
*CYP2D6*	c.100C>T	p.P34S	NS	rs1065852	likely benign		0.021	PA166156062; PharmVar	0.214	0.249	0.206
*CYP2D6*	c.985 + 39G>A		ncRNA_intronic	rs28371725			0.011	PA166156155	0.059	0.095	0.084
*CYP2E1*	c.1263T>C	p.F421F	synonymous	rs2515641	benign	0.03		PA166154017	0.856	0.887	0.880
*CYP2E1*			intronic	rs2070677		0.05		1f	0.856		0.867
*CYP4F12*			intronic	rs12460651		0.02	0.029	DFS & response	0.882		
***DFFB***			**intronic**	**rs4376673**		**0.04**	**0.047**	**DFS & response; PINES**	**0.909**	**0.947**	**0.901**
***DPYD***	**c.2194G>A**	**p.V732I**	**NS**	**rs1801160**	**likely_benign**	**0.02**		**PA166153647**	**0.052**	**0.047**	**0.045**
*DPYD*	c.1896T>C	p.F632F	synonymous	rs17376848	likely_benign	0.03		PA166153874; CADD	0.057	0.037	0.047
*DPYD*	c.496A>G	p.M166V	NS	rs2297595	drug_response			PA166153696; D	0.148	0.103	0.125
*DPYS*			intronic	rs2669429	not_provided		0.01	PA166157579	0.540		0.557
*ENOSF1*			intronic	rs2612083		0.01		1f	0.381	.	0.322
*EPHX2*	c.662G>A	p.R221Q	NS	rs751141	risk_factor	0.03		sequence in silico tools set	0.114	0.095	0.115
*EPHX2*	dist = 55		downstream	rs4149259		0.03		1f	0.167		
*ESR2*	c.*39G>A		UTR3	rs4986938			0.05	PA166154805	0.371	0.379	0.347
*GSTA1*	c.-9630T>C		UTR5	rs3957357			0.047	PA166157094	0.591		
*GSTA2*	c.335G>C	p.S112T	NS	rs2180314			0.017	PA166157020	0.592	0.590	0.581
*GSTP1*	c.313A>G	p.I105V	NS	rs1695	drug_response	0.05		PA166154249	0.329	0.319	0.320
*GSTP1*			intronic	rs762803		<0.001		1f; PINES	0.386	0.406	0.323
*IRS1*	c.*4476A>G		UTR3	rs2288587		0.05		1f	0.057		
***KCNAB1***			**intronic**	**rs2293194**		**0.04**	**0.013**	**DFS & response**	**0.476**		**0.515**
*MADD*			intronic	rs10501320		0.05		IW; PINES	0.281		
*NR5A2*	dist = 45		upstream	rs2816948		0.05	0.036	DFS & response; PINES	0.130		
*PIK3C2G*	c.2732C>T	p.P911L	NS	rs12312266		0.04		sequence in silico tools set	0.205	0.298	0.242
*PIP4K2B*			intronic	rs2075061		0.02		1f	0.605		0.592
*PPARA*	c.*5977G>A		UTR3	rs9626814			0.019	1d	0.101		.
*PPARG*	c.1347C>T	p.H449H	synonymous	rs3856806	likely_benign		0.046	PA166156388	0.120	0.125	0.133
*RALBP1*	c.*756G>A		UTR3	rs3322		0.03		1f	0.095	0.092	0.072
*RARB*	c.*1287T>G		UTR3	rs1058378			0.013	IW, miR-665	0.091		0.083
*RPTOR*	c.90T>C	p.F30F	synonymous	rs61750765		0.03		IW	0.238	0.126	0.142
*RRAGD*	c.*1105T>A		UTR3	rs1555403			0.019	1f	0.238		.
*SLC22A1*	c.1222A>G	p.M408V	NS	rs628031			0.028	PA166156933	0.600	0.592	0.605
***SLC28A3***	**c.338A>G**	**p.Y113C**	**NS**	**rs10868138**			**0.022**	**PA166157820**	**0.067**	**0.085**	**0.091**
*SLC2A1*	c.*462G>C		UTR3	rs4658	benign	0.01		PA166153544	0.210		0.178
*SLCO1A2*	c.-189_-188insA		UTR5	rs3834939		0.05		PA166163600	0.295		
*SLCO1C1*			intronic	rs34288910		0.03	0.028	DFS & response	0.144	0.128	0.152
*TUBB1*	c.*817G>C		UTR3	rs10485828		0.03		PA166155965	0.212		
*UGT2A1*	c.949G>A	p.G317R	NS	rs4148301			0.033	sequence in silico tools set	0.110	0.096	0.104

Validated variants (Section 2.2.2.) in bold. Variants rs3815583, rs1065852, and rs3322 were excluded due to technical failure. Footnotes: ^1^ NS = non-synonymous; ^2^ *p*-value provided for clinical associations; ^3^ Prediction based on combination of pharmacogenomic databases, e.g., PharmGKB (“PA” designation stands for specific diseases, genes and drugs in the database) and in silico individual tools, e.g., TargetScan (micro RNA target prediction), metaLR and metaSVM (D = deleterious), CADD (cut-off value ≥ 19), Nexus IW score under 0.01 (IW), PINES (*p*-value ≤ 0.05) or Regulome DB (score provided), and sequence in silico tools set (see Section 4 Materials and Methods and data provided in Table S4); ^4^ AF = non-reference allelic frequencies in the testing set; ^5^ Exome Aggregation Consortium (ExAc), allelic frequencies in European non-Finnish population; ^6^ National Center for Medical Genomics (NCMG), allelic frequencies in general Czech population.

**Table 6:** Clinical data of patients in the validation set.

Characteristics	Patients, *n* (%) ^1^
Age at diagnosis, mean ± S.D. ^2^ (years)	58.9 ± 12.5
Menopausal status	
Premenopausal	197 (25)
Postmenopausal	590 (75)
Missing data	18
Tumor size (pT)	
pTis	65 (8)
pT1	489 (62)
pT2	208 (27)
pT3	18 (2)
pT4	10 (1)
pTX	15
Lymph node metastasis (pN)	
Absent (pN0)	509 (67)
Present (pN1-3)	253 (33)
pNX	43
Pathological stage	
S0	61 (8)
SI	358 (47)
SII	282 (37)
SIII	67 (9)
Not determined	37
Histological type	
Invasive ductal carcinoma	598 (25)
Other type	197 (75)
Missing data	10
Pathological grade (G)	
G1	177 (23)
G2	385 (50)
G3	209 (27)
GX	34
Estrogen receptor status	
Positive	618 (77)
Negative	181 (23)
Missing data	6
Progesterone receptor status	
Positive	579 (73)
Negative	220 (27)
Missing data	6
Expression of HER2	
Positive	194 (24)
Negative	602 (76)
Missing data	9
Expression of Ki-67, mean ± S.D. ^2^ (%)	23.3 ± 22.6
Molecular subtype	
Luminal A	330 (41)
Luminal B	313 (39)
Triple negative	93 (12)
HER2	63 (8)
Missing data	6
Response to neoadjuvant cytotoxic therapy	
Complete or partial response	127 (75)
Stable disease or progression	43 (25)
Not applicable ^3^	635

Footnotes. ^1^ Number of patients with % in parentheses; ^2^ S.D. = standard deviation; ^3^ patients treated with adjuvant therapy without neoadjuvant cytotoxic therapy.

**Table 7:** Distribution of genotypes for variants assessed in the validation phase.

Gene	SNV	Genotypes ^1^			MAF ^2^	
		Common Homozygous	Heterozygous	Rare Homoz Ygous	Validation Set (*n* = 805)	Testing Set (*n* = 105)
*AKR7A2*	rs1043657	660	136	6	0.09	0.10
*TUBB1*	rs10485828	521	252	27	0.19	0.21
*MADD*	rs10501320	428	318	51	0.26	0.28
*RARB*	rs1058378	666	134	2	0.09	0.09
*SLC28A3*	rs10868138	681	108	8	0.08	0.07
*ABCB5*	rs11983326	409	332	59	0.28	0.28
*PIK3C2G*	rs12312266	462	290	44	0.24	0.21
*CYP4F12*	rs12460651	683	111	8	0.08	0.12
*RRAGD*	rs1555403	432	318	48	0.26	0.24
*GSTP1*	rs1695	366	355	76	0.32	0.33
*DPYD*	rs17376848	724	76	2	0.05	0.06
*DPYD*	rs1801160	729	74	0	0.05	0.05
*ABCA4*	rs1801466	678	117	4	0.08	0.11
*CYP2C9*	rs1934969	281	387	135	0.41	0.39
*AHRR*	rs2013782	312	389	97	0.37	0.41
*ABCB1*	rs2032583	639	158	6	0.10	0.11
*CYP2E1*	rs2070677	626	164	9	0.11	0.14
*CDA*	rs2072671	360	366	66	0.31	0.36
*PIP4K2B*	rs2075061	301	383	119	0.39	0.40
*BAK1*	rs210134	398	335	62	0.29	0.25
*ABCC1*	rs212091	598	186	14	0.13	0.18
*GSTA2*	rs2180314	251	409	141	0.43	0.41
*ATP7A*	rs2227291	499	262	37	0.21	0.26
*BIRC7*	rs2273487	227	405	161	0.46	0.49
*IRS1*	rs2288587	735	59	3	0.04	0.06
*KCNAB1*	rs2293194	229	373	198	0.48	0.48
*DPYD*	rs2297595	617	169	16	0.13	0.15
*CES1*	rs2307240	699	72	1	0.05	0.07
*CYP2E1*	rs2515641	627	166	9	0.11	0.14
*ENOSF1*	rs2612083	332	370	101	0.36	0.38
*DPYS*	rs2669429	246	421	135	0.43	0.46
*NR5A2*	rs2816948	619	165	11	0.12	0.13
*CYP2D6*	rs28371725	672	112	11	0.08	0.06
*ABCC6*	rs2856585	719	80	2	0.05	0.06
*ABCB8*	rs3214587	636	159	7	0.11	0.12
*SLCO1C1*	rs34288910	597	186	19	0.14	0.14
*ABCC1*	rs3743527	476	278	46	0.23	0.21
*AKT1*	rs3803304	407	317	64	0.28	0.29
*SLCO1A2*	rs3834939	366	360	77	0.32	0.30
*PPARG*	rs3856806	592	181	26	0.15	0.12
*GSTA1*	rs3957357	260	412	124	0.41	0.41
*UGT2A1*	rs4148301	644	147	10	0.10	0.11
*ABCC3*	rs4148413	499	236	43	0.21	0.17
*EPHX2*	rs4149259	569	212	22	0.16	0.17
*DFFB*	rs4376673	694	106	1	0.07	0.09
*SLC2A1*	rs4658	506	266	26	0.20	0.21
*ESR2*	rs4986938	329	367	103	0.36	0.37
*RPTOR*	rs61750765	575	208	17	0.15	0.24
*SLC22A1*	rs628031	302	372	122	0.39	0.40
*EPHX2*	rs751141	652	137	9	0.10	0.11
*CMPK1*	rs7543016	240	409	143	0.44	0.45
*GSTP1*	rs762803	289	402	108	0.39	0.39
*CES1*	rs76336259	714	87	0	0.05	0.06
*BLK*	rs922483	429	304	61	0.27	0.23
*PPARA*	rs9626814	627	163	9	0.11	0.10

Footnote: ^1^ Genotypes do not sum up to 805 due to missing data; ^2^ MAF = minor allele frequency.

**Table 8:** Validated variants significantly associating with the response of patients to neoadjuvant cytotoxic therapy in the validation phase.

Gene	SNV	Genotypes	Responders ^1^	Non-Responders ^1^	*p*-Value ^2^	*p*-Value Adj ^4^
*SLC28A3*	rs10868138				0.013	0.266
Solute Carrier Family 28 (Sodium-Coupled Nucleoside Transporter), Member 3—Nucleoside transporter with broad specificity for pyrimidine and purine nucleosides		Common homozygous	102	41		
		Rare allele ^3^	23	1		
*ATP7A*	rs2227291				<0.001	0.004
ATPase Copper Transporting Alpha—Copper transporter		Common homozygous	88	16		
		Heterozygous	29	24		
		Rare homozygote	9	2		
*KCNAB1*	rs2293194				0.003	0.030
Potassium Voltage-Gated Channel, Shaker-Related Subfamily, Beta Member 1—Pottasium channel		Common homozygous	42	9		
		Heterozygous	66	17		
		Rare homozygous	19	17		
*DFFB*	rs4376673				0.007	0.017
DNA Fragmentation Factor Subunit Beta—DNA fragmentation factor involved in apoptosis		Common homozygous	115	32		
		Rare allele ^3^	12	11		

Footnotes: ^1^ numbers of responders (complete or partial remission) or non-responders (stable or progressive disease); ^2^ *p*-value by the Pearson test; ^3^ in the absence of rare homozygotes in any of the compared groups, effect of rare allele was evaluated; ^4^ adjusted *p*-value by the multivariate logistic regression adjusted to disease stage.

**Table 9:** Validated associations of variants associating with DFS of patients treated with cytotoxic therapy according to their molecular subtypes.

Gene	SNV	Genotypes	Luminal A ^2^	Luminal B ^2^	HER2 ^2^	TN ^2,3^
*DPYD*	rs1801160		NS	**<0.001**	NS	**0.018**
Dihydropyrimidine Dehydrogenase—Pyrimidine catabolic enzyme		Common homozygous	90	**150**	33	**63**
		Rare allele ^1^	11	**16**	3	**3**
*DPYD*	rs17376848		NS	NS	**0.012**	NS
Dihydropyrimidine Dehydrogenase—Pyrimidine catabolic enzyme		Common homozygous	88	146	**33**	59
		Rare allele ^1^	13	20	**3**	7
*IRS1*	rs2288587		**0.002**	NS	NS	NS
Insulin Receptor Substrate 1—Protein mediating various cellular processes by insulin		Common homozygous	**93**	150	33	57
		Rare allele ^1^	**7**	14	3	8

Footnotes: ^1^ In the absence of rare homozygotes in any of the compared groups, effect of rare allele was evaluated; ^2^ *p*-value by the log-rank test and numbers of patients (significant associations are depicted in bold); ^3^ Triple negative; NS = Non-significant.

## Supplementary Materials

The following are available online at http://www.mdpi.com/2072-6694/10/12/511/s1, Figure S1: Groups of genes selected for the study, Figure S2: Detailed distribution of alterations in the studied groups of genes, Figure S3: Data curation from PharmGKB data files, Figure S4: Kaplan-Meier plots representing associations of variants with DFS of whole set of patients (rs12460651 and rs751141) or patients treated with hormonal without cytotoxic therapy (rs2075061), Table S1: Treatment regimens in the testing set of patients, Table S2: List of genes selected for the study, Table S3: List of associations between individual variants and survival or response of patients to cytotoxic therapy, Table S4: In silico predictions for variant prioritization. Table S5: Treatment regimens in the validation set of patients.
