# Supplementary material for: Use of Germline Genetic Variability for Prediction of Chemoresistance and Prognosis of Breast Cancer Patients
Source: Cancers (Basel). 2018 Dec 12;10(12):511. doi: 10.3390/cancers10120511 (PMC6316878; doi:10.3390/cancers10120511)
Supplement: Supplementary file 1 [file cancers-10-00511-s001.zip › cancers-393361-suppl-final.pdf]

Supplementary Materials: Use of Germline Genetic Variability for Prediction of Chemoresistance and Prognosis of Breast Cancer Patients

Viktor Hlavac, Maria Kovacova, Katerina Elsnerova, Veronika Brynychova, Renata Kozevnikovova, Karel Raus, Katerina Kopeckova, Sona Mestakova, David Vrana, Jiri Gatek, Pavel Ostasov, Radka Vaclavikova and Pavel Soucek

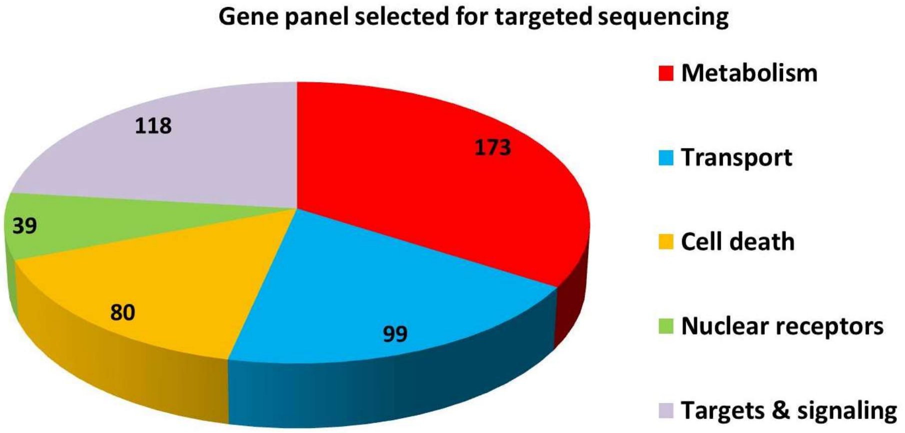

Figure S1. Groups of genes selected for the study.

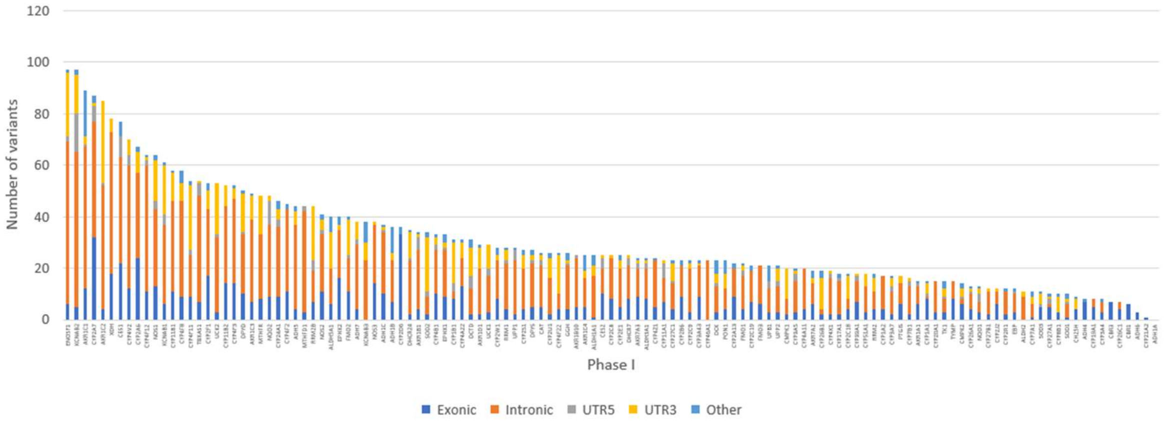

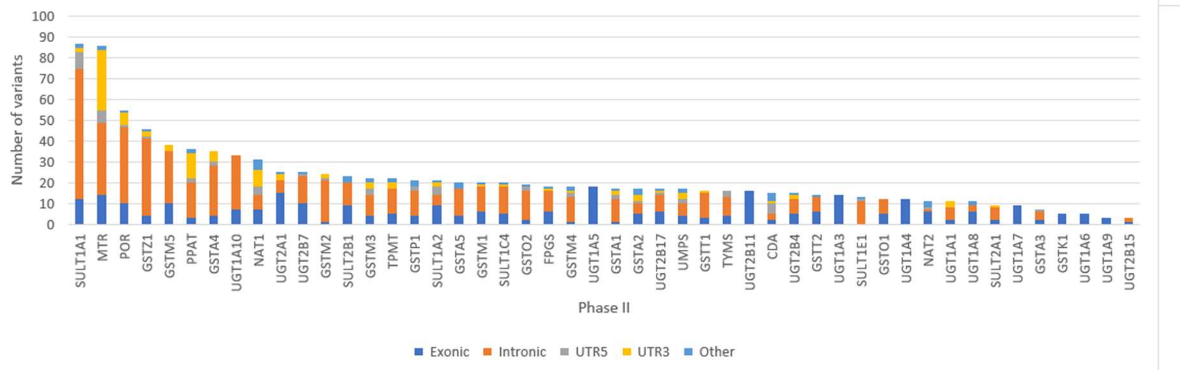

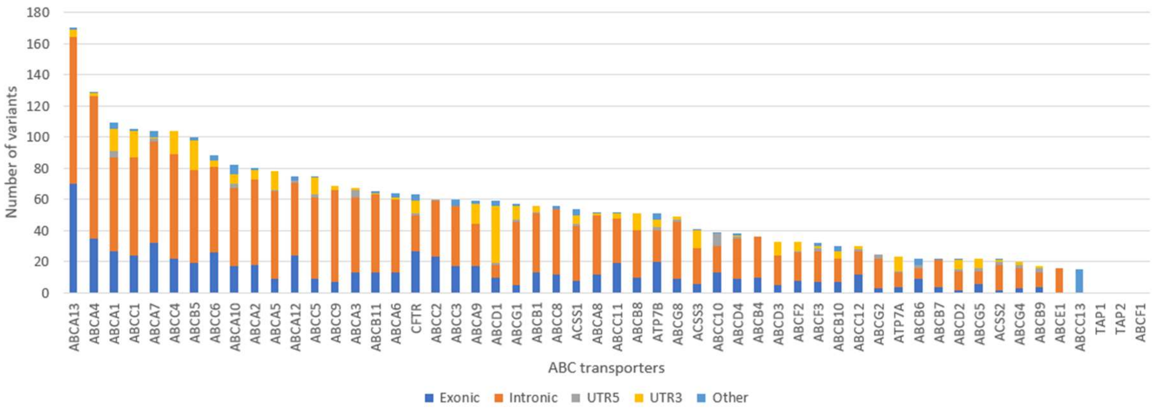
Number of variants
0
20
40
60
80
100
120
140
160
180
ABCA13
ABCA4
ABCA1
ABCC1
ABCA7
ABCC4
ABCB5
ABCC6
ABCA10
ABCA2
ABCA5
ABCA12
ABCC5
ABCC9
ABCA3
ABCB11
ABCA6
CFTR
ABCC2
ABCC3
ABCA9
ABCD1
ABCG1
ABCB1
ABCC8
ACSS1
ABCA8
ABCC11
ABCB8
ATP7B
ABCG8
ACSS3
ABCC10
ABCD4
ABCB4
ABCD3
ABCF2
ABCF3
ABCB10
ABCC12
ABCG2
ATP7A
ABCB6
ABCB7
ABCD2
ABCG5
ACSS2
ABCG4
ABCB9
ABCE1
ABCC13
TAP1
TAP2
ABCF1
ABC transporters
Exonic
Intronic
UTR5
UTR3
Other

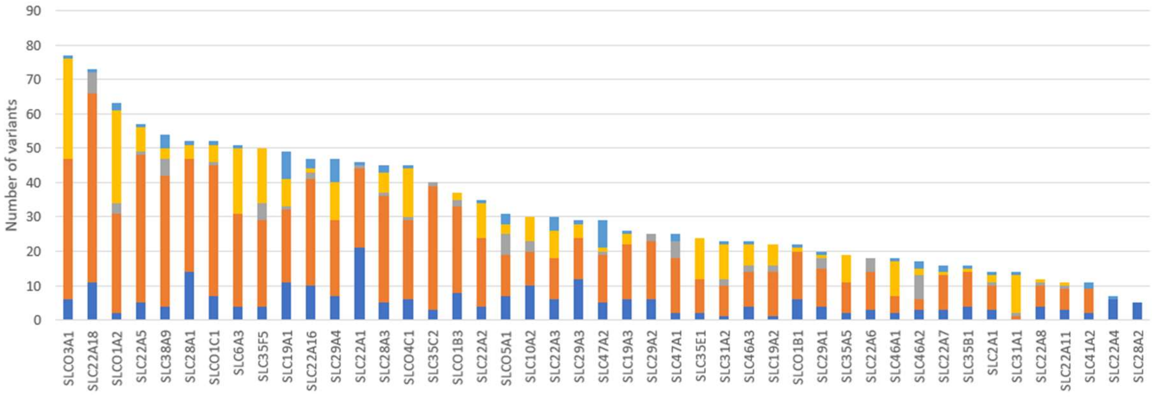
Number of variants
0
10
20
30
40
50
60
70
80
90
SLCO3A1
SLC22A18
SLCO1A2
SLC22A5
SLC38A9
SLC28A1
SLCO1C1
SLC6A3
SLC35F5
SLC19A1
SLC22A16
SLC29A4
SLC22A1
SLC28A3
SLCO4C1
SLC35C2
SLCO1B3
SLC22A2
SLCO5A1
SLC10A2
SLC22A3
SLC29A3
SLC47A2
SLC19A3
SLC29A2
SLC47A1
SLC35E1
SLC31A2
SLC46A3
SLC19A2
SLCO1B1
SLC29A1
SLC35A5
SLC22A6
SLC46A1
SLC46A2
SLC22A7
SLC35B1
SLC2A1
SLC31A1
SLC22A8
SLC22A11
SLC41A2
SLC22A4
SLC28A2

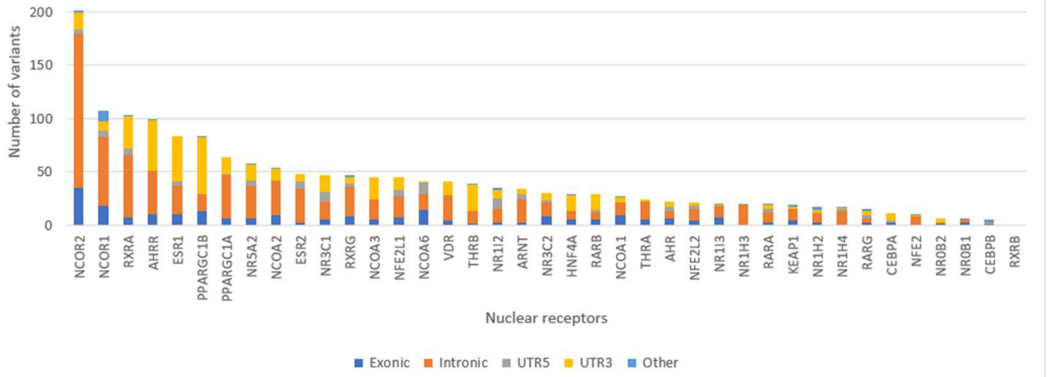
Number of variants
0
50
100
150
200
NCOR2
NCOR1
RXRA
AHRR
ESR1
PPARGC1B
PPARGC1A
NR5A2
NCOA2
ESR2
NR3C1
RXRG
NCOA3
NFE2L1
NCOA6
VDR
THRB
NR1I2
ARNT
NR3C2
HNF4A
RARB
NCOA1
THRA
AHR
NFE2L2
NR1I3
NR1H3
RARA
KEAP1
NR1H2
NR1H4
RARG
CEBPA
NFE2
NR0B2
NR0B1
CEBPB
RXRB
Nuclear receptors
Exonic
Intronic
UTR5
UTR3
Other

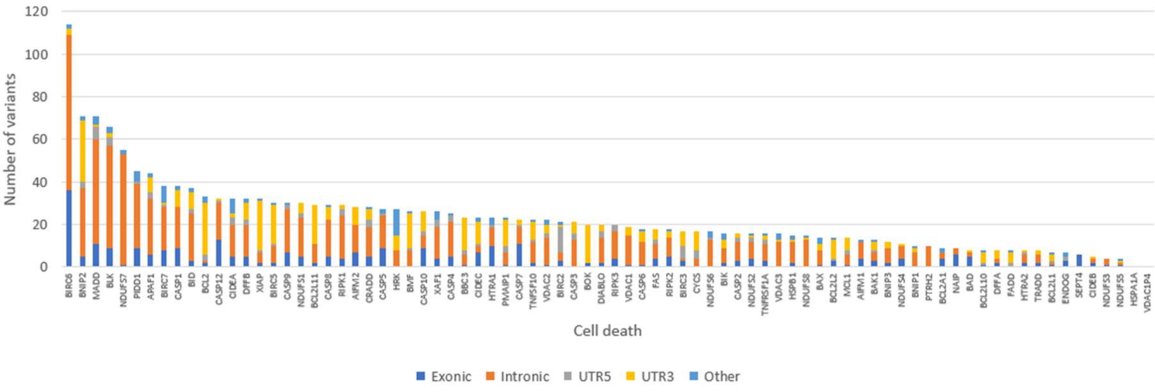
Number of variants
0
20
40
60
80
100
120
Cell death
Exonic
Intronic
UTR5
UTR3
Other

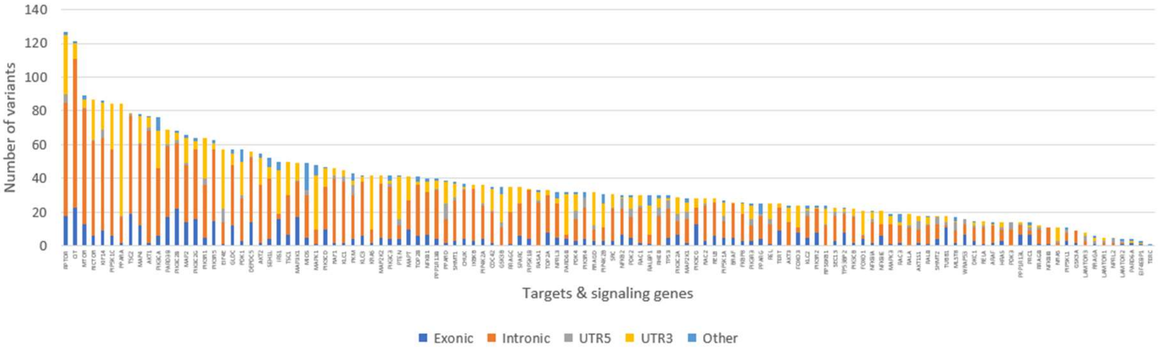

Figure S2. Detailed distribution of alterations in the studied groups of genes.

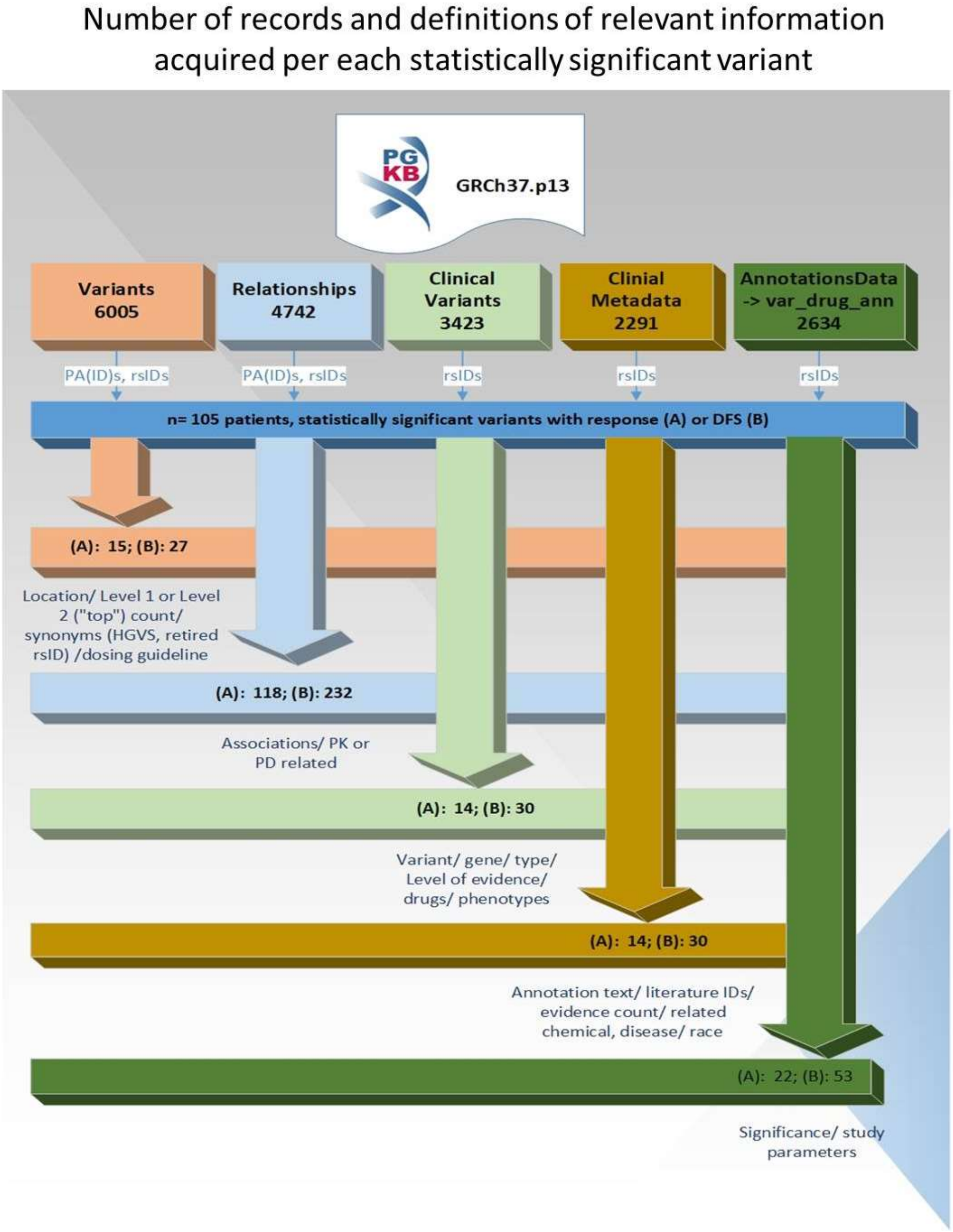

Figure S3. Data curation from PharmGKB data files.

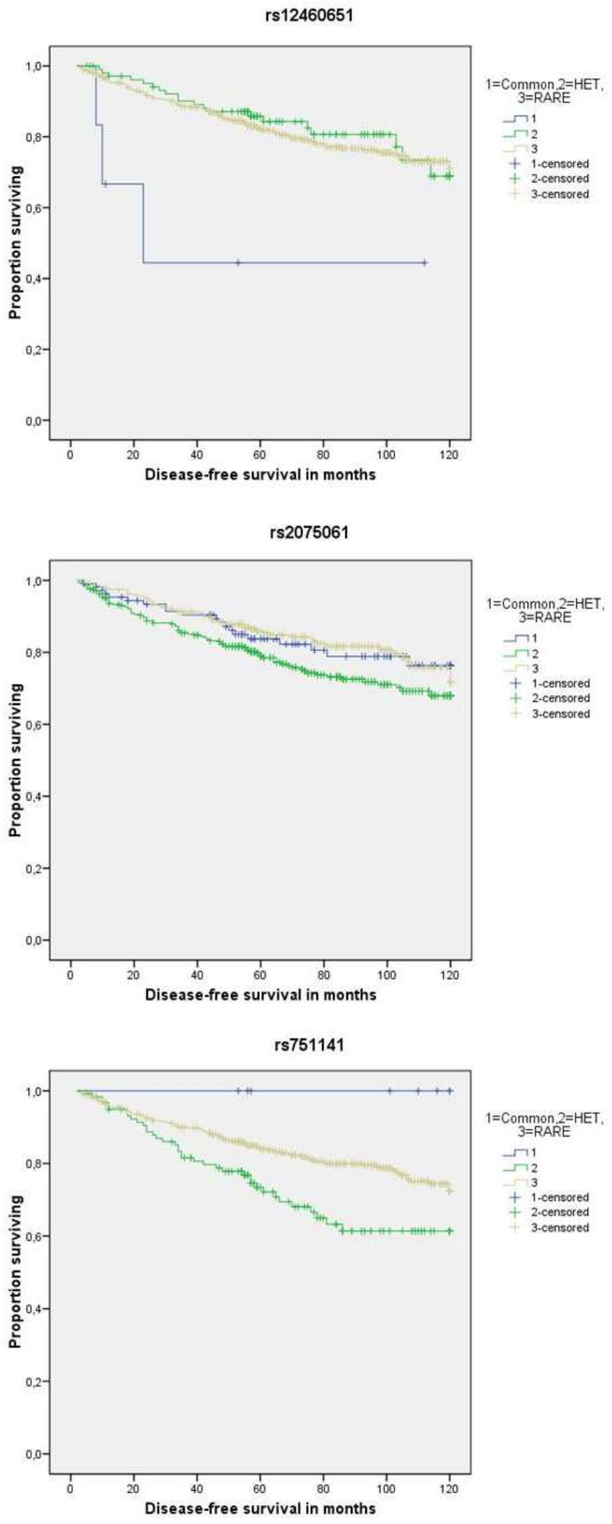

Figure S4. Kaplan-Meier plots representing associations of variants with DFS of whole set of patients (rs12460651 and rs751141) or patients treated with hormonal without cytotoxic therapy (rs2075061)

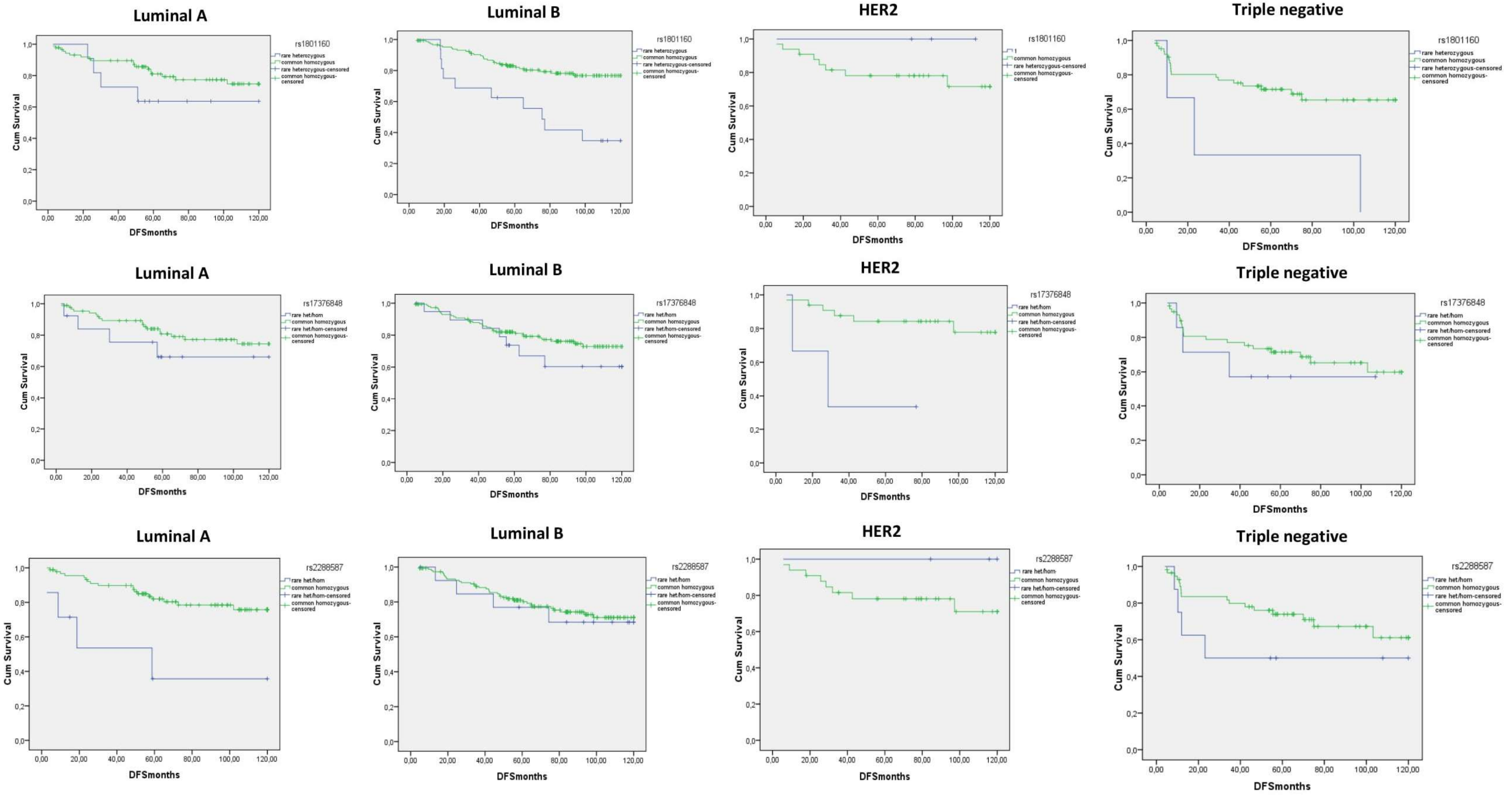

Figure S5. Kaplan-Meier plots representing associations of variants with DFS of patients stratified according to their molecular subtype.

Table S1. Treatment regimens in the testing set of patients.

A. Neoadjuvant cytotoxic therapy (*n* = 68).

Drugs (regimen)	*N*
anthracycline with cyclophosphamide (and 5-fluorouracil)	22
anthracycline with taxane	6
anthracycline with cyclophosphamide (and 5-fluorouracil) followed with taxane	39
other (1× taxane with vinorelbine)	1

B. Adjuvant cytotoxic therapy (*n* = 32) [1.]

Drugs (regimen)	*N*
anthracycline with cyclophosphamide (and 5-fluorouracil)	15
anthracycline with cyclophosphamide followed with taxane	15
taxane monotherapy	1
other (1× capecitabine with vinorelbine)	1

Footonote: [1] In five patients no cytotoxic therapy was used.

Tables S2–S4 are in other separated files.

Table S5. Treatment regimens in the validation set of patients.

A. Neoadjuvant therapy (*n* = 180) [1.]

Drugs (regimen)	*N*
anthracycline with cyclophosphamide (and 5-fluorouracil)	55
anthracycline with taxane	12
anthracycline with cyclophosphamide (and 5-fluorouracil) followed with taxane	95
taxane monotherapy	2
other (1× taxane with vinorelbine, 15× hormonal therapy)	16

B. Adjuvant cytotoxic therapy (*n* = 300).

Drugs (regimen)	N
anthracycline with cyclophosphamide (and 5-fluorouracil)	143
anthracycline with taxane	3
anthracycline with cyclophosphamide (and 5-fluorouracil) followed with taxane	63
taxane monotherapy	75
Other [2] (10× CMF, 2× CMF followed with taxane, 1× GEMOX followed with taxane, 1× monotherapy with vinorelbine, 1× monotherapy with anthracycline, 1× monotherapy with capecitabine)	16

C. Adjuvant hormonal therapy (*n* = 585) [3.]

Drugs (regimen)	N
tamoxifen monotherapy	299
aromatase inhibitors monotherapy	181
tamoxifen and aromatase inhibitors switch	101
other (3x monotherapy with Zoladex)	3

In 54 patients targeted therapy with herceptin was used in monotherapy (*n* = 22) or in combination with cytotoxic or hormonal therapy (*n* = 32). In 83 patients no hormonal or cytotoxic therapy was used in neoadjuvant or adjuvant setting. For 84 patients information about therapy was not available.

Footnotes: [1] adjuvant cytotoxic therapy was used in 88 patients pretreated with neoadjuvant therapy (all patients included in part B); [2] CMF = cyclophosphamide, methotrexate and 5-fluorouracil;

GEMOX = gemcitabine with oxaliplatin; [3]adjuvant hormonal therapy without cytotoxic agents was used in 312 patients (the distribution of treatment regimens was comparable to C).

© 2018 by the authors. Licensee MDPI, Basel, Switzerland. This article is an open access article distributed under the terms and conditions of the Creative Commons Attribution (CC BY) license (http://creativecommons.org/licenses/by/4.0/).
